# Exploring Seasonal and Circadian Rhythms in Structural Traits of Field Maize from LiDAR Time Series

**DOI:** 10.34133/2021/9895241

**Published:** 2021-09-06

**Authors:** Shichao Jin, Yanjun Su, Yongguang Zhang, Shilin Song, Qing Li, Zhonghua Liu, Qin Ma, Yan Ge, LingLi Liu, Yanfeng Ding, Frédéric Baret, Qinghua Guo

**Affiliations:** ^1^Plant Phenomics Research Centre, Academy for Advanced Interdisciplinary Studies, Collaborative Innovation Centre for Modern Crop Production Co-Sponsored by Province and Ministry, Jiangsu Key Laboratory for Information Agriculture, Nanjing Agricultural University, Nanjing 210095, China; ^2^Jiangsu Provincial Key Laboratory of Geographic Information Science and Technology, International Institute for Earth System Sciences, Nanjing University, Nanjing, Jiangsu 210023, China; ^3^State Key Laboratory of Vegetation and Environmental Change, Institute of Botany, Chinese Academy of Sciences, Beijing 100093, China; ^4^University of Chinese Academy of Sciences, Beijing 100049, China; ^5^National Technique Innovation Center for Regional Wheat Production/Key Laboratory of Crop Ecophysiology, Ministry of Agriculture, Nanjing Agricultural University, Nanjing, 210095 Jiangsu, China; ^6^Department of Forestry, Mississippi State University, Mississippi State 39759, USA; ^7^Environnement Méditerranéen et Modélisation des Agro-Hydrosystèmes (EMMAH), Institut National de la Recherche Agronomique, Unité Mixte de Recherche 1114 Domaine Saint-Paul, Avignon Cedex 84914, France; ^8^Department of Ecology, College of Environmental Sciences, and Key Laboratory of Earth Surface Processes of the Ministry of Education, Peking University, Beijing 100871, China

## Abstract

Plant growth rhythm in structural traits is important for better understanding plant response to the ever-changing environment. Terrestrial laser scanning (TLS) is a well-suited tool to study structural rhythm under field conditions. Recent studies have used TLS to describe the structural rhythm of trees, but no consistent patterns have been drawn. Meanwhile, whether TLS can capture structural rhythm in crops is unclear. Here, we aim to explore the seasonal and circadian rhythms in maize structural traits at both the plant and leaf levels from time-series TLS. The seasonal rhythm was studied using TLS data collected at four key growth periods, including jointing, bell-mouthed, heading, and maturity periods. Circadian rhythms were explored by using TLS data acquired around every 2 hours in a whole day under standard and cold stress conditions. Results showed that TLS can quantify the seasonal and circadian rhythm in structural traits at both plant and leaf levels. (1) Leaf inclination angle decreased significantly between the jointing stage and bell-mouthed stage. Leaf azimuth was stable after the jointing stage. (2) Some individual-level structural rhythms (e.g., azimuth and projected leaf area/PLA) were consistent with leaf-level structural rhythms. (3) The circadian rhythms of some traits (e.g., PLA) were not consistent under standard and cold stress conditions. (4) Environmental factors showed better correlations with leaf traits under cold stress than standard conditions. Temperature was the most important factor that significantly correlated with all leaf traits except leaf azimuth. This study highlights the potential of time-series TLS in studying outdoor agricultural chronobiology.

## 1. Introduction

Plant growth rhythm depicts biological and physiological behaviors of plants, such as “tree sleeping” at night [1–3], which is controlled by an endogenous timing mechanism (i.e., biological clock) and/or exogenous environment [4, 5]. Plant growth rhythm exists in most organisms [6]. Exploring the mechanism of plant growth rhythm is important for understanding the response and adaptation strategies of different plants to the ever-changing environment [4, 7].

Plant growth rhythm has been extensively and accurately studied in plant physiology [7–9], but has rarely been explored in structure. Plant structural rhythm is regulated by both the biological clock and environment, which can directly capture plant structure dynamics and their relationships with plant functions [10]. It is becoming a promising direction to understand plant rhythm which is now more easily accessible with the development of sensing technologies, computer vision, and deep learning algorithms [11, 12].

Light detection and ranging (LiDAR), an emerging active sensing technology less influenced by the natural illumination conditions, measures the three-dimensional (3D) structure of a plant with high precision and accuracy. LiDAR has been recognized as a cutting-edge tool in quantifying plant structure across different environments [13, 14]. During the last several decades, LiDAR has been extensively used to extract tree height [15–18], leaf area index [19], and plant volume [20]. Meanwhile, multitemporal LiDAR data has been used to monitor plant dynamics, such as tree growth, biomass change, and carbon dynamics in forestry [21–23], horticulture [4], and agriculture [24]. These studies proved the feasibility of LiDAR to characterize long-term (e.g., seasonal and yearly) structural rhythms under field conditions [3, 25–27].

Recently, a few studies have tried to use hypertemporal LiDAR data to study short-term (e.g., hourly) structural rhythm in trees. For example, Puttonen et al. [2] used terrestrial laser scanning (TLS) to monitor the nocturnal movement of birch branches and foliage. They observed that the vertical tree height at sunrise was lower than that at the last sunset and named this pattern “tree sleeping.” They further demonstrated that the largest movements of tree crown and branches appeared around sunrise [28]. However, some studies demonstrated different subcircadian growth rhythms [29] and not all trees show the same structural circadian rhythms [3, 30]. These efforts indicated that structural rhythms are complicated and need to be further explored.

These investigations using multitemporal and hypertemporal LiDAR applied to trees [3, 28, 29] question whether long-term and short-term rhythms can be also observed in crops under field conditions. Unlike using LiDAR in characterizing structural dynamics in trees, LiDAR application in crops has much more challenges, including (1) crops have a small size that requires high-quality LiDAR data, (2) the main component of crops is the leaves that are easily affected by environmental conditions (e.g., wind and temperature), and (3) leaves are more complex and variable in shape and curvature thus more difficult to identify and characterize. Additionally, previous studies focused mainly on circadian rhythm under nonstressed conditions [2, 3, 28, 29]. Circadian rhythms under stressful environments, such as cold stress, are also of great interest.

In this study, we aim to quantify the seasonal and circadian rhythms in maize structural traits at the plant and leaf levels under standard (plants grow naturally without environmental stress) and cold stress conditions in field environments from TLS time series. Specific questions are targeted. (1) Can we quantify the seasonal rhythms of structural traits? (2) Can we quantify the circadian rhythms under standard and cold stress conditions? (3) What are the possible relationships between the environmental factors and the circadian rhythms of structural traits under standard and cold stress conditions?

## 2. Data and Methods

### 2.1. Study Area

**Figure 1:**
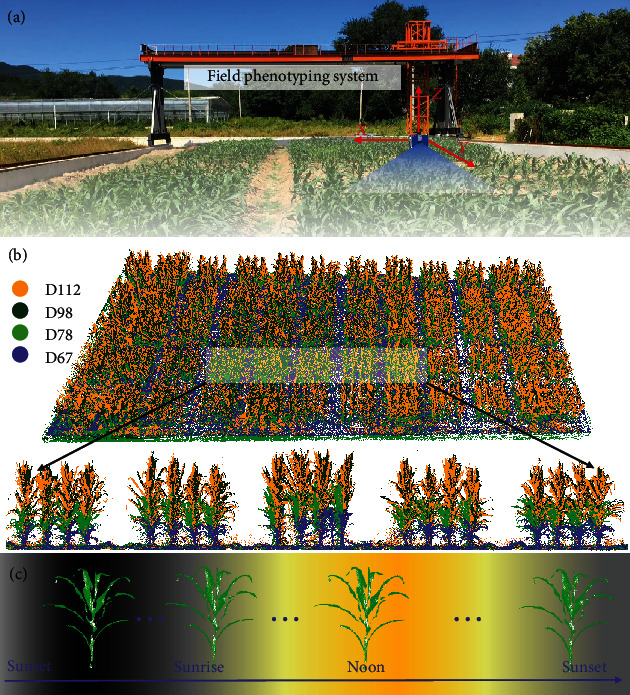
Study area and LiDAR data collection. (a) The field phenotyping gantry system supporting the LiDAR. (b) LiDAR data was collected at four key periods on 67, 78, 98, and 112 days after planting (i.e., D67, D78, D98, and D112). (c) Example of LiDAR data collected around every two hours throughout a whole day from one sunset to the next sunset.

The study area was located at the Xiangshan breeding base of the Institute of Botany, Chinese Academy of Sciences (40°8′10^″^N, 116°10′46^″^E), Beijing, China. The planting area was around 600 m^2^ (Figure 1(a)). The annual average precipitation was 572.38 mm, and the annual average temperature was 12.96°C. We planted two rounds of maize (*Zea mays* L.) on Mar. 25, 2019 (Experiment 1, hereafter Exp. 1), and Aug. 25, 2019 (Experiment 2, hereafter Exp. 2), respectively. A total of 55 plots were planted in the two experiments. The plot size was 3 m × 3 m, and the row and column spacing between adjacent plots was both 1 m. In each plot, plants were planted at an interval of 0.6 m in both row and column directions. Exp. 1 grew well with the standard cultivation and management, which was used to collect data for studying seasonal and circadian rhythms under standard conditions. Exp. 2 did not have a complete growth period due to cold stress, and it was only used for studying the circadian rhythm.

### 2.2. Data Collection

In this study, TLS data were collected using a high-throughput phenotyping platform, Crop3D, developed by the authors' team (Figure 1(a)) [31, 32]. A LiDAR sensor integrated with the system can move in *x*, *y*, and *z* directions accurately, flexibly, and automatically according to a predefined program that ensures to record consistent time-series data. The LiDAR was mounted at the height of 0.5 m above the canopy, and the moving speed was set at 0.05 m/s in both *x* and *y* directions. In this study, the system was operated at a multistation mode, which means that the sensor scanned data at a fixed location instead of scanning while moving. This multistation mode can acquire data with around 2 mm resolution at a 10 m distance on an object with 90% reflectance. Besides, the wavelength of the LiDAR sensor is 1550 nm, which is out of the range of visible light mainly used by plant growth. The average power of the emitted beam is 500 mW, and the laser emits nanosecond pulses that have little influence on plant growth. Additionally, to minimize the influence of environmental factors on scanning, we only scanned data on carefully selected days that meet the following requirements: (1) days belong to key growth periods and (2) weather condition was stable with no rain, no water condensation, and no wind/occasional gusts (human observation) during data acquisition.

Exp. 1 was used to acquire data for studying seasonal and circadian rhythms under standard conditions. Data for studying seasonal rhythm were scanned at four key growth periods, including jointing, bell-mouthed, heading, and maturity periods, which were 67, 78, 98, and 112 days after planting (hereafter D67, D78, D98, and D112). Data for studying circadian rhythm were scanned at the late bell-mouthed stage around every 2 hours from sunset on June 22, 2019, to sunset on June 23, 2019 (D89-D90). The moments of sunset, sunrise, and noon (Table 1) were referred to https://www.timeanddate.com/. In this study, the moments of sunset (D89), sunrise (D90), noon (D90), and sunset (D90) were 19:46, 04:46, 12:16, and 19:46, respectively. The scanning moments were trying to cover the moments of sunset, sunrise, and noon. However, to avoid interference from environmental factors (e.g., gusts), the interval of scanning moments was not exactly but around 2 hours. Finally, data were collected at 16 moments from D89 to D90, including 19:49, 21:48, 23:48, 01:48, 3:48, 4:48, 6:48, 8:48, 10:48, 12:00, 12:48, 14:48, 16:48, 18:48, 19:48, and 21:48 (Table 1).

Exp. 2 was only used to acquire data at the bell-mouthed stage for studying circadian rhythms under early cold stress. During this period, the plant did not die but showed certain symptoms of chilling injury, such as frostbite on the edge of the leaf. Data for studying circadian rhythm were scanned around every 2 hours from sunset on Nov. 9, 2019, to sunset on Nov. 10, 2019 (D76-D77). The moments of sunset, sunrise, noon, and data scanning were determined using the same method in Exp. 1 (Table 1). In this study, the moments of sunset (D76), sunrise (D77), noon (D77), and sunset (D77) were 17:04, 06:52, 11:58, and 19:03, respectively. Finally, data were scanned at 14 moments from D76 to D77, including 18:00, 20:00, 22:00, 22:30, 24:00, 02:00, 04:00, 06:00, 08:00, 10:00, 12:00, 14:00, 16:00, and 18:00 (Table 1).

In addition to LiDAR data, this study recorded environmental data, including the incoming photosynthetically active radiation (PAR, *μ*mol·m^−2^·s^−1^), temperature (*T*, °C), and relative humidity (RH, %), every half hour through the whole growing season. PAR was recorded using SPN1 Sunshine Pyranometer (Delta-T Devices Ltd., UK). *T* and RH were recorded using an in situ weather station (Decagon Devices Inc., USA).

### 2.3. Data Preprocessing

**Figure 2:**
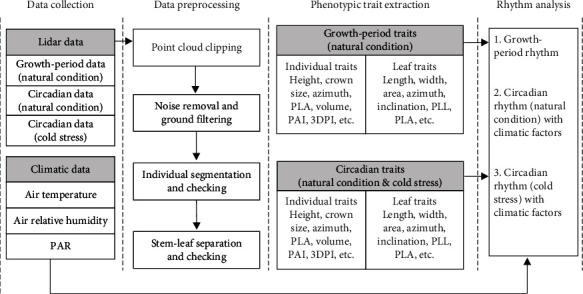
Scheme for processing collected LiDAR data and environmental data for phenotypic trait extraction and structural rhythm analysis. PAR, PLA, PAI, 3DPI, PLL, and PLA represent photosynthetically active radiation, projected leaf area, plant area index, three-dimensional profile index, projected leaf length, and projected leaf area, respectively.

TLS data were processed with a series of standard steps, including clipping, noise removal, filtering, normalization, individual plant segmentation, and stem-leaf segmentation (Figure 2). The time-series single-station TLS data acquired at the same location were used to study seasonal and circadian rhythms to minimize the influence of registration error that was around 2 mm according to Jin et al. [32].

The single station data was first manually clipped to obtain data in an area of 3 m × 3 m where the point density is high (around 1,000,000 points/m^2^). The clipped data were preprocessed with a statistical outlier removal method integrated in LiPlant software (the Green Valley International®). The noise-removed data was filtered and normalized using a digital-terrain-model-based method described in Jin et al. [32]. The normalized points were segmented to extract individual plants using a deep learning-based method [33] integrated in LiPlant. Visual inspection and manual revision were conducted to improve the accuracy of the segmented plants. These plants were further processed to extract individual leaves and stems using a voxel-based deep learning method [34] with interactive corrections in LiPlant. Ears at heading and mature stages were manually removed and were not included in the analysis.

In Exp. 1, a total of 30 plants were randomly selected from the segmented results from 10 single station data. These plants include 231, 317, 361, and 324 leaves, respectively, for the four periods considered. A few leaves fell in the last (mature) period, resulting in a decrease in the number of leaves. The average value of the several traits investigated was computed for the four periods and was used for studying seasonal rhythm (Table 1). In addition, a total of 10 randomly selected plants with 109 leaves from 160 single stations, covering 16 moments, were used for studying the circadian rhythms under standard conditions. In Exp. 2, a total of 10 randomly selected individual plants with 73 leaves from 140 single stations, covering 14 moments, were used for studying the circadian rhythm under cold stress.

### 2.4. Extraction of Structural Traits

Previous studies mainly extracted height quantiles as structural traits to study the tree dynamics [2, 3]. In addition to these height quantiles, we derived more meaningful biophysical traits, such as the leaf area, and investigate their dynamics at both the plant and the leaf levels.

At the plant level, nine traits were extracted, including maximum height (*H*_max_, m), mean height (*H*_mean_, m), 99% height quantile (*H*_99_, m), crown size, plant azimuth angle, projected leaf area (PLA, m^2^), volume, projected area index (PAI, m^2^/m^2^), and three-dimensional profile index (3DPI). The plant azimuth angle was defined as the angle between the maximum eigenvector of the plant and the north direction on the vertical projection plane (Supplementary Fig. 1a). Definitions and formulas of the other traits can be found at Jin et al. [32].

At the leaf level, nine leaf traits were extracted, including leaf length, maximum leaf width, mean leaf width, leaf height, leaf area, projected leaf length (PLL, m), PLA, leaf inclination, and leaf azimuth. Of which, the definitions and formulas of leaf length, leaf width, leaf area, and leaf inclination can be found at Jin et al. [35]. Leaf height, PLL, and PLA were defined in Jin et al. [32]. Leaf azimuth was defined as the angle between the maximum eigenvector of a leaf and the north direction on the vertical projection plane (Supplementary Fig. 1b).

### 2.5. Statistic and Correlation Analysis

To show whether TLS can characterize the seasonal rhythm in structural traits, Tukey's multicomparison method was used to test the difference among the measurement dates at the 0.05 confidence level. Besides, because the circadian rhythm is driven by local environmental signals (e.g., light, temperature, and air humidity) [30, 36], the Pearson correlation coefficient and *p* value were used to analyze the relationship between these environmental factors and the circadian rhythms in plant and leaf structural traits under standard and cold stress conditions. The circadian rhythm observation time and the climate data recording time were not always fully synchronized. The raw half-hour climate data were therefore interpolated using a cubic spline function to predict the climate data corresponding to the circadian rhythm observation.

## 3. Results

### 3.1. Seasonal Rhythm of Plant and Leaf Structural Traits

**Figure 3:**
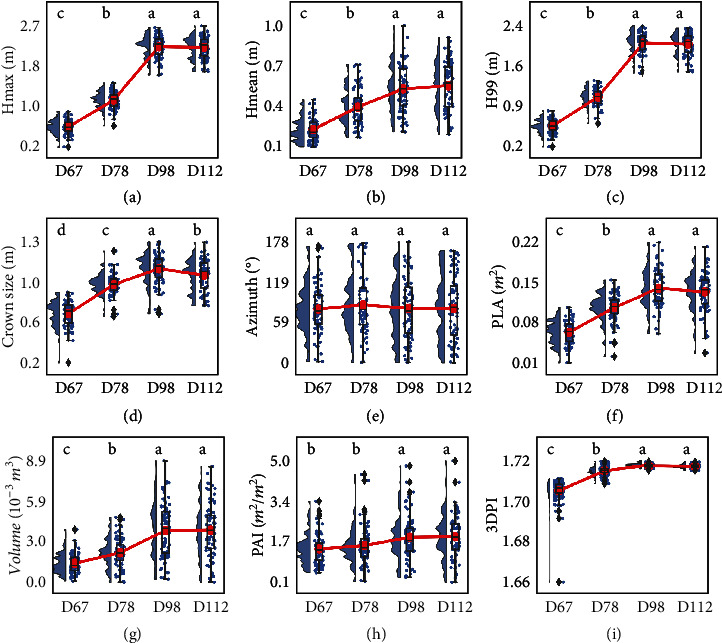
Seasonal rhythms of plant structural traits, including (a) *H*_max_, (b) *H*_mean_, (c) *H*_99_, (d) crown size, (e) azimuth, (f) PLA, (g) volume, (h) PAI, and (i) 3DPI. D67, D78, D98, and D112 represented the four growth periods on 67, 78, 98, and 112 days after planting. The lowercase letters on each subfigure represented the results of multiple comparisons of each trait in different growth periods at the 0.05 confidence level using Tukey's multicomparison method.

At the individual plant level, results show that TLS can describe the seasonal rhythms of several structural traits of maize (Figure 3). Height-related traits (i.e., *H*_max_, *H*_mean_, and *H*_99_), PLA, volume, and 3DPI increased significantly in the first three stages and became stable at the mature stage. Crown size showed a significant increase in the first three stages but decreased significantly at the mature stage. PAI was stable at the first two and the last two stages, but it showed significant growth from the second (bell-mouthed) to the third (heading) stage. Interestingly, the azimuth angle of individual plants was stable throughout all stages.

**Figure 4:**
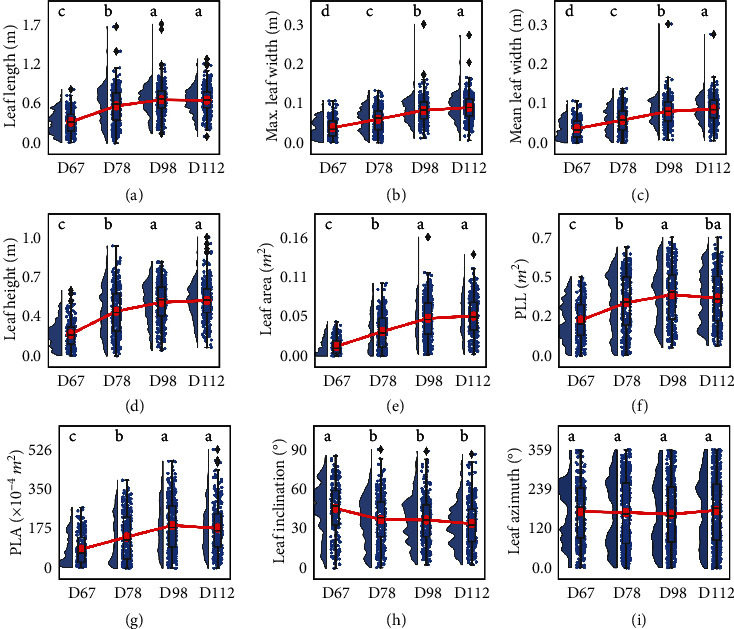
Seasonal rhythms of leaf structural traits, including (a) leaf length, (b) max. leaf width, (c) mean leaf width, (d) leaf height, (e) leaf area, (f) PLL, (g) PLA, (h) leaf inclination, and (i) leaf azimuth. D67, D78, D98, and D112 represent four key periods on 67, 78, 98, and 112 days after planting. The lowercase letters on each subfigure represented the results of multiple comparisons of each trait in different growth periods at the 0.05 confidence level using Tukey's multicomparison method.

At the leaf level (Figure 4), results showed that TLS can also quantify the seasonal rhythms of several traits. Results show that the dynamics at the leaf level were more diverse than that at the plant level. Leaf length, leaf height, leaf area, PLL, and PLA increased significantly in the first three stages and became stable at the mature stage. Maximum and mean leaf width showed a significant increase throughout all stages. Leaf inclination angle decreased significantly at the first stage, and it remained unchanged at the following stages. The leaf azimuth angle was stable throughout all stages, consistently with the plant azimuth.

### 3.2. Circadian Rhythm in Structural Traits under Standard Condition

The growth of the several traits during the season (Figure 3) results from a significant daily increase: for example, 6 cm growth can be observed for *H*_max_ between two adjacent sunsets (Supplementary Fig. 2). Therefore, the daily increase contributing to the seasonal growth should be eliminated before studying the circadian rhythms. We thus calculated the average growth rate per hour of each trait between D78 and D112 according to Figures 3 and 4. We then corrected the raw variation of the traits during the day by subtracting the average hourly seasonal growth at the time of observation (Supplementary Fig. 2 and 3).

**Figure 5:**
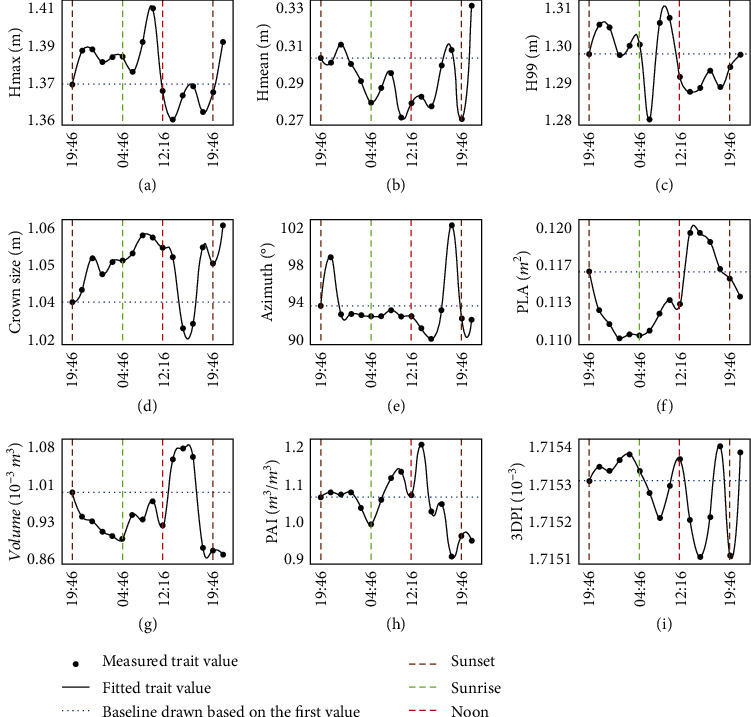
Circadian rhythms in structural traits of maize at the individual plant level under standard conditions, including (a) *H*_max_, (b) *H*_mean_, (c) *H*_99_, (d) crown size, (e) azimuth, (f) PLA, (g) volume, (h) PAI, and (i) 3DPI. The *x*-axis is the measured moments from June 22 to June 23, 2019. Each point indicated an averaged trait value of the selected 10 individual plants.

At the plant level (Figure 5), results showed that TLS can quantify the circadian rhythms of several structural traits of maize. Different circadian patterns are observed depending on the traits. (1) The height-related traits, *H*_max_ and *H*_99_, increased at night, while *H*_mean_ and other height quantiles (Supplementary Fig. 4) decreased at night. Further, these height-related traits all showed large fluctuations during the daytime. (2) Crown size increased throughout the whole day except in the afternoon. (3) PLA, volume, and PAI decreased at night, increased in the morning, and decreased in the afternoon. (4) Azimuth and 3DPI showed no obvious rhythm.

**Figure 6:**
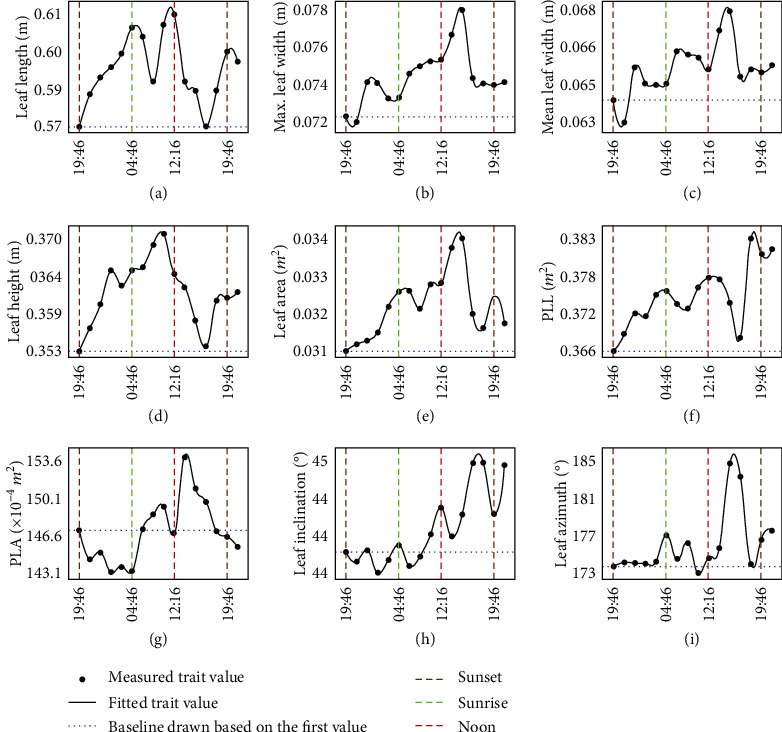
Circadian rhythms in structural traits of maize at the leaf level under standard conditions, including (a) leaf length, (b) max. leaf width, (c) mean leaf width, (d) leaf height, (e) leaf area, (f) PLL, (g) PLA, (h) leaf inclination, and (i) leaf azimuth. The *x*-axis is the measured moments from June 22 to June 23, 2019. Each point indicated an averaged trait value of the selected 109 leaves.

TLS measurements can also quantify the circadian rhythms of several leaf-level structural traits that appeared to be more diverse than at the plant level (Figure 6). Results showed that (1) leaf length, maximum leaf width, mean leaf width, leaf height, leaf area, and PLL increased from sunset to noon and decreased in the afternoon with some fluctuations in the changing trend. (2) PLA decreased at night, increased in the morning, and decreased in the afternoon, consistently with plant-level observations. (3) Leaf azimuth is almost stable, consistently with the plant azimuth. Leaf inclination is also stable except for the increase in the afternoon. Both for the plant- and leaf-level traits, the rhythms were more fluctuant during daytime than during the night.

### 3.3. Circadian Rhythm in Structural Traits of Maize under Cold Stress Condition

**Figure 7:**
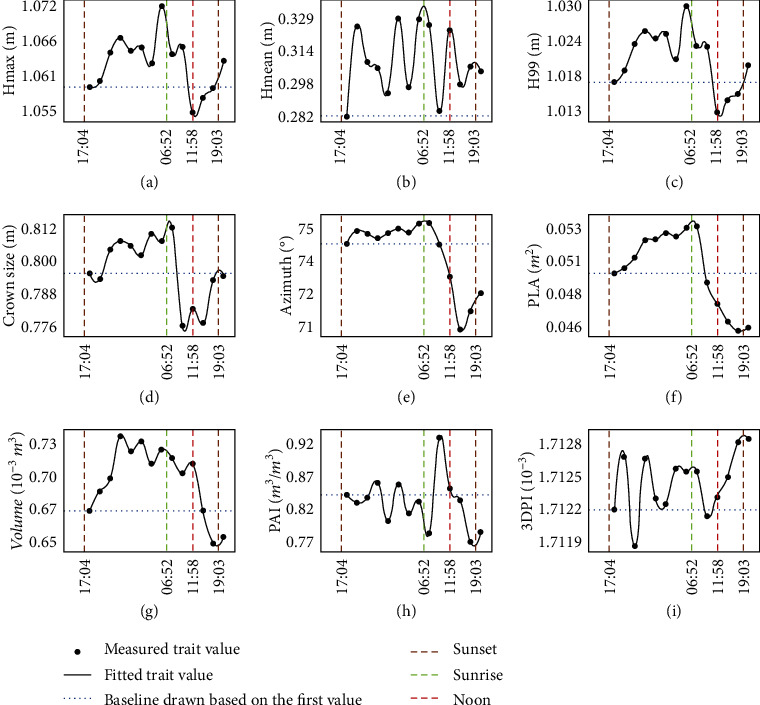
Circadian rhythms in structural traits of maize at the individual plant level under cold stress, including (a) *H*_max_, (b) *H*_mean_, (c) *H*_99_, (d) crown size, (e) azimuth, (f) PLA, (g) volume, (h) PAI, and (i) 3DPI. The *x*-axis is the measurement moment from Nov. 9 to Nov. 10, 2019. Each point indicated an averaged trait value of the selected 10 individual samples.

At the plant level (Figure 7), TLS can quantify the circadian rhythms in multiple structural traits of maize under cold stress. Different traits showed different circadian rhythms. (1) *H*_max_, *H*_99_ (and other height quantiles in Supplementary Fig. 5), and crown size increased during the whole day except for an obvious decrease in the morning. (2) Azimuth, PLA, and volume increased at night and decreased during the daytime. (3) *H*_mean_, PAI, and 3DPI showed no regular rhythm.

**Figure 8:**
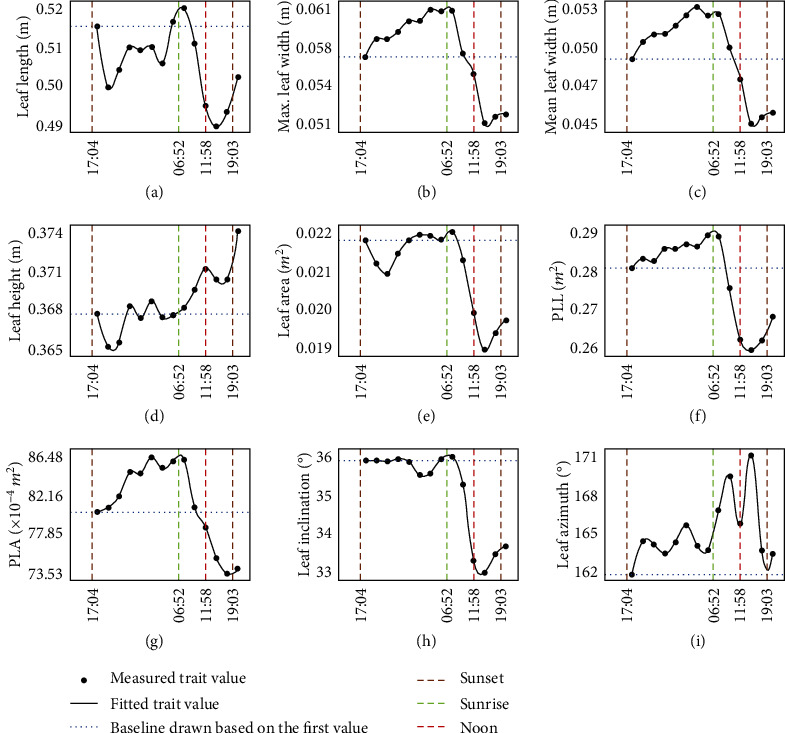
Circadian rhythms in structural traits of maize at the leaf level under cold stress, including (a) leaf length, (b) maximum leaf width, (c) mean leaf width, (d) leaf height, (e) leaf area, (f) PLL, (g) PLA, (h) leaf inclination, and (i) leaf azimuth, at leaf level under cold stress. The *x*-axis is the measurement moment from Nov. 9 to Nov. 10, 2019. Each point indicated an averaged trait value of the selected 73 leaves.

At the leaf level (Figure 8), results showed that TLS can quantify the circadian rhythms in multiple structural traits of maize under cold stress. The results showed different rhythms that (1) maximum leaf width, mean leaf width, PLL, and PLA showed a similar pattern that increased at night and decreased during the daytime. (2) Leaf height and leaf inclination were stable at night. However, leaf inclination decreased during the daytime, while leaf height increased. (3) Leaf azimuth increased at night and in the morning, but it decreased in the afternoon. Leaf length and leaf area were fluctuating at night, decreased in the morning, and increased in the afternoon.

Overall, TLS can quantify the circadian rhythm in structural traits at both individual plant and leaf levels under cold stress. Many individual traits (e.g., *H*_max_ and *H*_99_) and leaf traits (i.e., length, width, and area) showed a similar pattern that increased at night and decreased during the daytime. PLA increased at night and decreased during the daytime at both plant and leaf levels, which was contrary to the circadian rhythm of PLA under standard conditions. Additionally, most rhythms under cold stress (e.g., *H*_max_, *H*_99_, crown size, azimuth, PLA, and volume) were clearer (less fluctuation) than circadian rhythms under standard conditions.

## 4. Discussion

### 4.1. Importance of High-Accuracy and High-Throughput Terrestrial LiDAR Data

High throughput and high accuracy are the two most important prerequisites for studying the seasonal and circadian rhythms of structural traits of maize plants and leaves. Previous efforts have demonstrated that TLS can provide high point density, making accurate phenotyping applicable at multiple biological levels [14]. At the plant level, numerous traits have been accurately extracted from TLS data, such as crop height [33, 37, 38], crown size [35], and PAI [24, 39]. With the advancement of point cloud processing algorithm (e.g., deep learning) [34, 40–43], phenotypes at organ levels were extracted, such as branch length [44] and leaf inclination [35]. These technological advances guarantee the accuracy of rhythm studies in structural traits.

Besides high accuracy, high throughput is also an important but still challenging requirement for studying structural rhythms. Previous studies have tried to acquire LiDAR on a daily scale for studying phenological changes in forest ecosystems [25, 27]. Recent studies have demonstrated that TLS data can be acquired with even shorter intervals (e.g., hourly or dozens of minutes) for quantifying the overnight movement of tree branches and foliage [2, 28]. These studies resulted in large data sets which were very demanding in storage and processing capacity [21], explaining why they focused on a limited set of samples. In our study, dozens of individuals and hundreds of leaves were tracked and analyzed for studying both the seasonal and circadian structural rhythms. Further, although the scanning interval was similar to previous studies, this is the first study conducted in the agriculture field, as well as experiments under both natural and cold stress conditions.

### 4.2. Seasonal Rhythm in Structural Traits of Maize

Unlike previous works focusing on tree height dynamics, this study provided a more comprehensive description of the seasonal rhythm by considering more structural traits of maize. At the plant level, most findings are consistent with our experience that structural traits (e.g., height-related traits, PLA, volume, and 3DPI) grow during the vegetative growth periods and become stable at the mature stage. The pattern of volume and 3DPI has rarely been reported because it is difficult to measure these two traits using traditional methods, such as measuring with a ruler or optical image. Further, the seasonal rhythms revealed some new findings. For example, the crown size decreased significantly at the mature stage, which is almost consistent with the trends of PLA (Figure 3) and is similar to the patterns found in Maddonni and Otegui [45]. PAI only showed significant growth from the second (bell-mouthed) to the third (heading) stage, similar to the growth pattern reported in Gao et al. [46], which may be caused by the increasing photosynthesis capacity during the reproductive period. The azimuth angle after D67 was stable, which may result from the competition between plants for light [47–49]. However, it would be worth exploring whether the azimuth is changing before D67. All these findings prove that TLS described accurately the seasonal rhythm. This can be further exploited to better understand crop functioning and for making decisions for crop management.

At the leaf level, the seasonal dynamics have rarely been studied because they have higher requirements on data quality and processing methods. Our results showed that the leaf-level seasonal rhythms were more diverse. This is possibly explained by the variability in the rhythm of the leaves of a different order [50, 51], which are subjected to a different microclimate including light and temperature [52]. The rhythm of most traits (e.g., leaf length, leaf height, leaf area, PLL, and PLA) met our expectations: they grew first and became stable at the mature stage. The decrease of leaf inclination angle between D67 (jointing) and D78 (bell-mouthed) may be caused by the competition for the resources (e.g., space and light) at the early stage [53]. Additionally, the structural rhythm of azimuth and PLA traits was consistent with that of the plant-level rhythm, confirming that some leaf-level trait rhythm can be used to indicate the structural rhythm of an individual plant [54].

### 4.3. Circadian Rhythm in Structural Traits of Maize under Natural and Cold Stress Conditions

Studying circadian rhythm is much more difficult because plants may have a slight movement during a short interval [30]. Meanwhile, because plant movement is easily influenced by environmental factors including wind, previous studies have mainly focused on structural dynamics at night [2, 29]. By contrast, this study tried to study circadian rhythms by carefully collected data during windless “time windows.”

Under standard conditions, most findings are easy to understand. Some findings are interesting and deserve further exploration. For example, *H*_mean_ decreased at night, which is consistent with previous findings about “tree sleeping” at night [2, 3]. However, *H*_max_ and *H*_99_ increased at night, which indicates that crop height reduction (i.e., “sleep”) may mainly occur in the lower layer that is consistent with the dynamics of height percentile in Zlinszky et al. [3]. PLA, volume, and PAI decreased at night, increased in the morning, and decreased in the afternoon, which seems to be regulated by temperature and/or photosynthetic rates [55]. Previous studies have also pointed out that the plants near the morning and evening move greatly [2, 28, 56].

Besides, we can see that the rhythm of the day is not as obvious as the rhythm of the night because environmental conditions are more complex during the daytime as mentioned in previous studies [2]. We found that the circadian rhythms of PLA and azimuth angle at the leaf level are similar to those at the plant level which was also discussed earlier with the seasonal rhythm at both levels. However, for the other traits, the circadian rhythm at the leaf level is generally more clear (less fluctuation) than that at the plant level. This may be the rhythm that occurs at the organ level may be neutralized at the individual plant level due to the environmental entrainment [6]. This explains why some studies select parts of the plant (e.g., branch) as the observation target instead of the whole plant to study the structural rhythms [2].

Under a cold stress environment, traits showed clearer (less fluctuation) circadian rhythms (e.g., *H*_max_, *H*_99_, crown size, azimuth, PLA, and volume) than under standard conditions. This may be because plants harden their tissues to prevent freeze injury, making circadian rhythm less disturbed [57]. Most leaf traits (i.e., *H*_max_, *H*_99_, leaf length, leaf width, and leaf area) showed a similar pattern that increased at night and decreased during the daytime under cold stress (Figure 8). However, PLA increased at night and decrease during the daytime at both individual plant and leaf levels, which is contrary to the rhythm under standard conditions. The increase of PLA at night under cold stress may be caused by the movement of leaf azimuth angle (Figure 8(i)), while the possible reason for the decrease of PLA during the daytime is the decrease of leaf inclination angle (Figure 8(h)). Additionally, the circadian rhythm of the night is clearer (less fluctuation) than the rhythm of the day. Leaf-level circadian rhythm is also clearer than that at the plant level. These patterns are consistent with patterns found under standard conditions. Finally, it should be noted that these rhythms found in the early stages of cold stress may not be universal, because different types of plants respond differently under different degrees of chilling injury [58].

Previous studies have found that plant seasonal rhythms are regulated by environmental factors such as photoperiod, temperature, and water balance [30, 59] and the plants tending to adapt to their local environment [36]. In this study, we tried to understand the influence of the environment on the circadian rhythm in structural traits of the crop. It seems that the circadian rhythm at the plant level is less affected by the environment under standard conditions (Table 2), but is easily affected by temperature and RH under cold stress. Besides, the circadian rhythm at the leaf level was also less affected by the environment under standard conditions and was mainly affected by temperature and RH, especially temperature, under cold stress (Table 3). Additionally, circadian rhythm may be more complicated under cold stress because different environmental factors may have significant correlations with traits at the same time under cold stress [60, 61].

In summary, TLS can capture circadian rhythm in structural traits of maize under natural and cold stress conditions at both the plant and leaf levels. The structural rhythms at night are more clear (less fluctuation) than the rhythm during the daytime. Leaf-level circadian rhythms are more clear than those at the plant level. Circadian rhythms under cold stress are more clear than those rhythms under standard conditions.

### 4.4. Contributions and Future Works

This study demonstrated that TLS can be used to analyze the seasonal and circadian rhythms of structural traits of maize at the plant and leaf levels under natural and cold stress conditions. To our knowledge, this is the first effort that uses TLS to study the structural rhythms of maize. Besides, this study analyzed the structural rhythm not only at night but also during the daytime. Moreover, structural rhythm under cold stress was studied and compared with standard conditions, which contributed to better understanding of the structural response of crops to different environments.

However, some challenges still need to be improved in future works. First, we did not set a rigid marker as a reference in the time-series analysis to quantify the observational error during LiDAR data collection. Puttonen et al. [2] used the movement of spheres attached to the tree branch as a reference, which has little influence on the branch movement. However, it is very hard to attach a reference for maize leaves. Second, the circadian rhythm that is mixed with the seasonal growing (Section 3.2) was corrected using a linear correction method. However, the daily growth of maize may be nonlinear. This may be the reason why the end point of the circadian rhythm curve did not arrive at the same level as the start point of some traits. Actually, the slight variation of environmental conditions and phenotypic trait extraction accuracy may also have inevitable influence on the circadian rhythms. Third, the frequency of observation for studying the seasonal rhythm should be increased in the future. Although most of the current traits showed structural rhythms, increasing the frequency of observations would offer the potentials to discover more seasonal patterns. For example, there is no significant change in the azimuth angle after D67; it is worth exploring whether it has changed before D67 to adapt to the environment [62]. Fourth, because the observation on the hourly scale of day and night is very time-consuming and laborious, this study and other similar studies all do not have repeated observations on consecutive days [2, 3, 28, 30, 63]. In the future, solving the difficulties of high-throughput observation, big data management, and data processing of TLS applications will provide a better foundation for high-frequency structural rhythm studies. Fifth, studying the structural rhythms of different varieties is helpful for exploring the plant endogenous timing mechanism and discovering genes related to the biological clock, etc. Besides, it is interesting to quantify the impact of environmental tress (e.g., cold) on the normal growth rhythms if we know the mechanism of the biological clock. Finally, the seasonal and circadian rhythms were studied using TLS data only; multisource remote sensing data are expected to use, such as using hyperspectral, thermal, and chlorophyll fluorescence to track the physiological rhythms [12, 64]. Meanwhile, simultaneous measurement of changes in plant physiology (e.g., stomatal movement), biochemistry, and metabolite indicators have important prospects for explaining changes in plant structural rhythms [7, 65, 66]. It will also be of great significance for mining genes that regulate circadian rhythms by studying the different patterns of different species.

## 5. Conclusion

The research of plant rhythms has a long history, but the use of LiDAR for studying structural rhythms is indeed a novel and interesting topic in recent years. This study explored the potential of TLS in the study of seasonal and circadian rhythms at the plant and leaf levels under standard and cold stress conditions. The seasonal rhythms in structural traits are clear and consistent with our expectations. Some structural traits such as azimuth and PLA show good consistency between plant- and leaf-level rhythms. However, the overall seasonal rhythms at the leaf level are more diverse, such as the decreasing of leaf inclination angle between the jointing stage and the bell-mouthed stage. By contrast, circadian rhythms are more complicated. Circadian rhythms of some traits (e.g., *H*_max_ and *H*_99_) under cold stress and standard conditions are opposite. Additionally, we found environmental factors have more significant correlations with leaf trait rhythms under cold stress than standard conditions, especially air temperature under cold stress conditions. This study highlights the potential of time-series TLS in studying crop chronobiology in the outdoor environment. By combining structural rhythms with plant biology theories, it will allow improving our understanding of the mechanism driving plant rhythms and the survival strategy of plants in the context of environmental changes.

## Figures and Tables

**Table 1:** Description of collected LiDAR data.

Planting date	Data collection time	Number of selected individual samples	Number of selected leaf samples	Treatment	Usage
Mar. 25, 2019	Daytime on May 31, June 11, July 11, and July 15, 2019	80	1233 leaves from 30 randomly selected individual plants, including 231, 317, 361, and 324 leaves in the four periods, respectively	Standard condition	Seasonal rhythm at plant and leaf levels

Mar. 25, 2019	16 scans at 19:49, 21:48, 23:48, 01:48, 3:48, 4:48, 6:48, 8:48, 10:48, 12:00, 12:48, 14:48, 16:48, 18:48, 19:48, and 21:48 from June 22 to June 23, 2019	10	109 leaves segmented from 10 randomly selected individual plants	Standard condition	Circadian rhythm at plant and leaf levels
Aug. 25, 2019	14 scans at 18:00, 20:00, 22:00, 22:30, 24:00, 02:00, 04:00, 06:00, 08:00, 10:00, 12:00, 14:00, 16:00, and 18:00 from Nov. 9 to Nov. 10, 2019	10	73 leaves segmented from 10 randomly selected individual plants	Cold stress	

**Table 2:** The relationships denoted by the Pearson correlation coefficient and *p* value between environmental factors (photosynthetically active radiation (PAR), temperature (*T*), and relative humidity (RH)) and circadian rhythms in structural traits at the plant level under standard and cold stress conditions. PLL and PLA represent projected leaf length and projected leaf area, respectively.

Traits	Standard conditions			Cold stress		
	PAR	RH	*T*	PAR	RH	*T*
*H*_max_	-0.08	**0.53**	**-0.56**	-0.87	0.42	**-0.62**
*H*_mean_	-0.39	-0.08	-0.09	0.27	0.04	-0.1
*H*_99_	0.06	0.2	-0.38	0.68	**0.56**	-0.75
Crown size	**0.67**	0.45	-0.3	0.67	**0.59**	**-0.66**
Azimuth	-0.13	-0.23	0.07	0.14	0.66	**-0.94**
PLA	-0.47	**-0.84**	**0.91**	0.41	0.69	**-0.93**
Volume	-0.39	-0.44	0.48	0.48	**0.55**	-0.74
PAI	-0.03	0.09	-0.09	0.48	0.11	-0.26
3DPI	0	0.36	-0.49	-0.31	-0.33	0.4

Normal represents *p* > 0.05; bold represents *p* < 0.05; underline represents *p* < 0.01; bold-underline represents *p* < 0.001.

**Table 3:** The relationship denoted by the Pearson correlation coefficient and *p* value between environmental factors (photosynthetically active radiation (PAR), temperature (*T*), and relative humidity (RH)) and circadian rhythm in structural traits at leaf level under standard and cold stress growth. PLL and PLA represent projected leaf length and projected leaf area, respectively.

Traits	Standard conditions			Cold stress		
	PAR	RH	*T*	PAR	RH	*T*
Leaf length	0.17	0.64	-0.42	-0.71	0.36	-0.71
Max. leaf width	0	-0.16	0.44	0.24	**0.65**	**-0.92**
Mean leaf width	0.13	-0.09	0.38	0.45	**0.66**	**-0.93**
Leaf height	0.8	0.65	-0.42	-0.1	-0.7	**0.79**
Leaf area	0.16	0.01	0.31	-0.67	**0.64**	**-0.92**
PLL	0.35	0.02	0.21	0.25	0.72	**-0.95**
PLA	-0.05	**-0.54**	0.73	0.39	**0.65**	**-0.9**
Inclination	-0.21	**-0.53**	0.69	0.13	0.73	**-0.95**
Azimuth	-0.17	-0.3	0.45	0.28	-0.42	0.31

Normal represents *p* > 0.05; bold represents *p* < 0.05; underline represents *p* < 0.01; bold-underline represents *p* < 0.001.

## Data Availability

The data could be given upon reasonable request from the corresponding author.

## Supplementary Materials

Supplementary Materials Supplementary Figure 1: the definition of azimuth angle at the individual plant and leaf levels. Figure 2: circadian rhythms mixed with self-growing in structural traits of maize at the individual plant level under standard conditions, including (a) *H*_max_, (b) *H*_mean_, (c) *H*_99_, (d) crown size, (e) azimuth, (f) PLA, (g) volume, (h) PAI, and (i) 3DPI. The *x*-axis is the measured moments from June 22 to June 23, 2019. Figure 3: circadian rhythms mixed with self-growing in structural traits of maize at the leaf level under standard conditions, including (a) leaf length, (b) max. leaf width, (c) mean leaf width, (d) leaf height, (e) leaf area, (f) PLL, (g) PLA, (h) leaf inclination, and (i) leaf azimuth. The *x*-axis is the measured moments from June 22 to June 23, 2019. Figure 4: circadian rhythms in structural traits of maize at the individual plant level under standard conditions, including (a) *H*_90_, (b) *H*_80_, (c) *H*_70_, (d) *H*_60_, (e) *H*_50_, (f) *H*_40_, (g) *H*_30_, (h) *H*_20_, and (i) *H*_10_. Figure 5: circadian rhythms in structural traits of maize at the individual plant level under cold stress, including (a) *H*_90_, (b) *H*_80_, (c) *H*_70_, (d) *H*_60_, (e) *H*_50_, (f) *H*_40_, (g) *H*_30_, (h) *H*_20_, and (i) *H*_10_.
