# Supplementary material for: Exploring Seasonal and Circadian Rhythms in Structural Traits of Field Maize from LiDAR Time Series
Source: Plant Phenomics. 2021 Sep 6;2021:9895241. doi: 10.34133/2021/9895241 (PMC8441379; doi:10.34133/2021/9895241)
Supplement: Supplementary Materials — Supplementary Figure 1: the definition of azimuth angle at the individual plant and leaf levels. Figure 2: circadian rhythms mixed with self-growing in structural traits of maize at the individual plant level under standard conditions, including (a) Hmax, (b) Hmean, (c) H99, (d) crown size, (e) azimuth, (f) PLA, (g) volume, (h) PAI, and (i) 3DPI. The x-axis is the measured moments from June 22 to June 23, 2019. Figure 3: circadian rhythms mixed with self-growing in structural traits of maize at the leaf level under standard conditions, including (a) leaf length, (b) max. leaf width, (c) mean leaf width, (d) leaf height, (e) leaf area, (f) PLL, (g) PLA, (h) leaf inclination, and (i) leaf azimuth. The x-axis is the measured moments from June 22 to June 23, 2019. Figure 4: circadian rhythms in structural traits of maize at the individual plant level under standard conditions, including (a) H90, (b) H80, (c) H70, (d) H60, (e) H50, (f) H40, (g) H30, (h) H20, and (i) H10. Figure 5: circadian rhythms in structural traits of maize at the individual plant level under cold stress, including (a) H90, (b) H80, (c) H70, (d) H60, (e) H50, (f) H40, (g) H30, (h) H20, and (i) H10. [file 9895241.f1.docx]

**Supplementary**


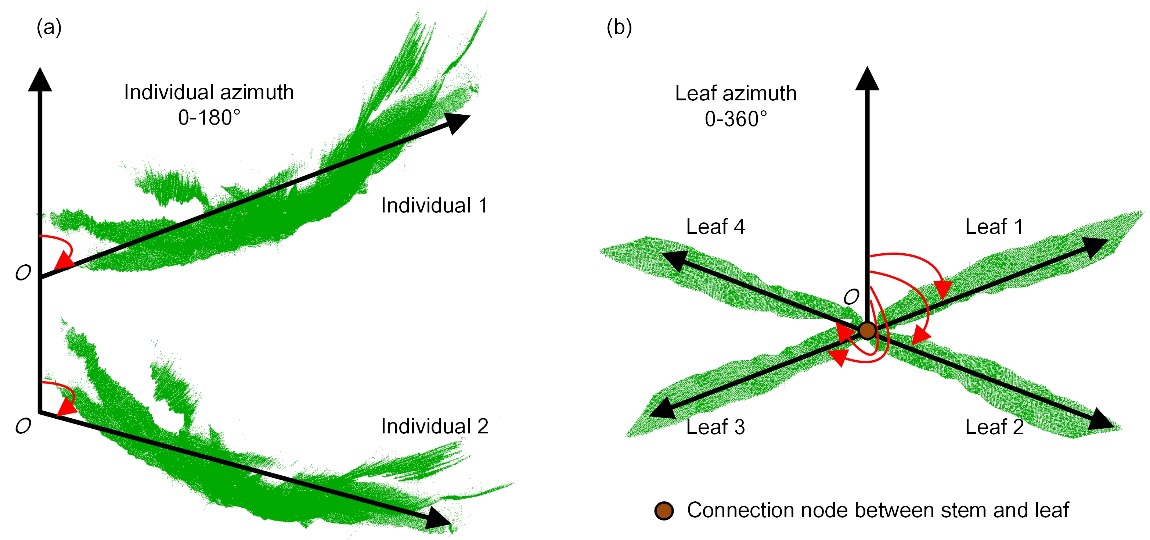


**Figure 1.** the definition of azimuth angle at the individual plant and leaf levels. (a) the individual azimuth angle is defined as the angle between the maximum eigenvector of a maize plant and the north direction on the vertical projection plane. The origin (*O*) of coordinate axes is selected as the leftmost point of the individual plant. The value of individual azimuth is between 0° and 180°. (b) Leaf azimuth is defined as the angle between the maximum eigenvector of a leaf and the north direction on the vertical projection plane. The origin (*O*) of coordinate axes is selected as the connection node between a leaf and a stem object. The value of leaf azimuth is between 0° and 360°.


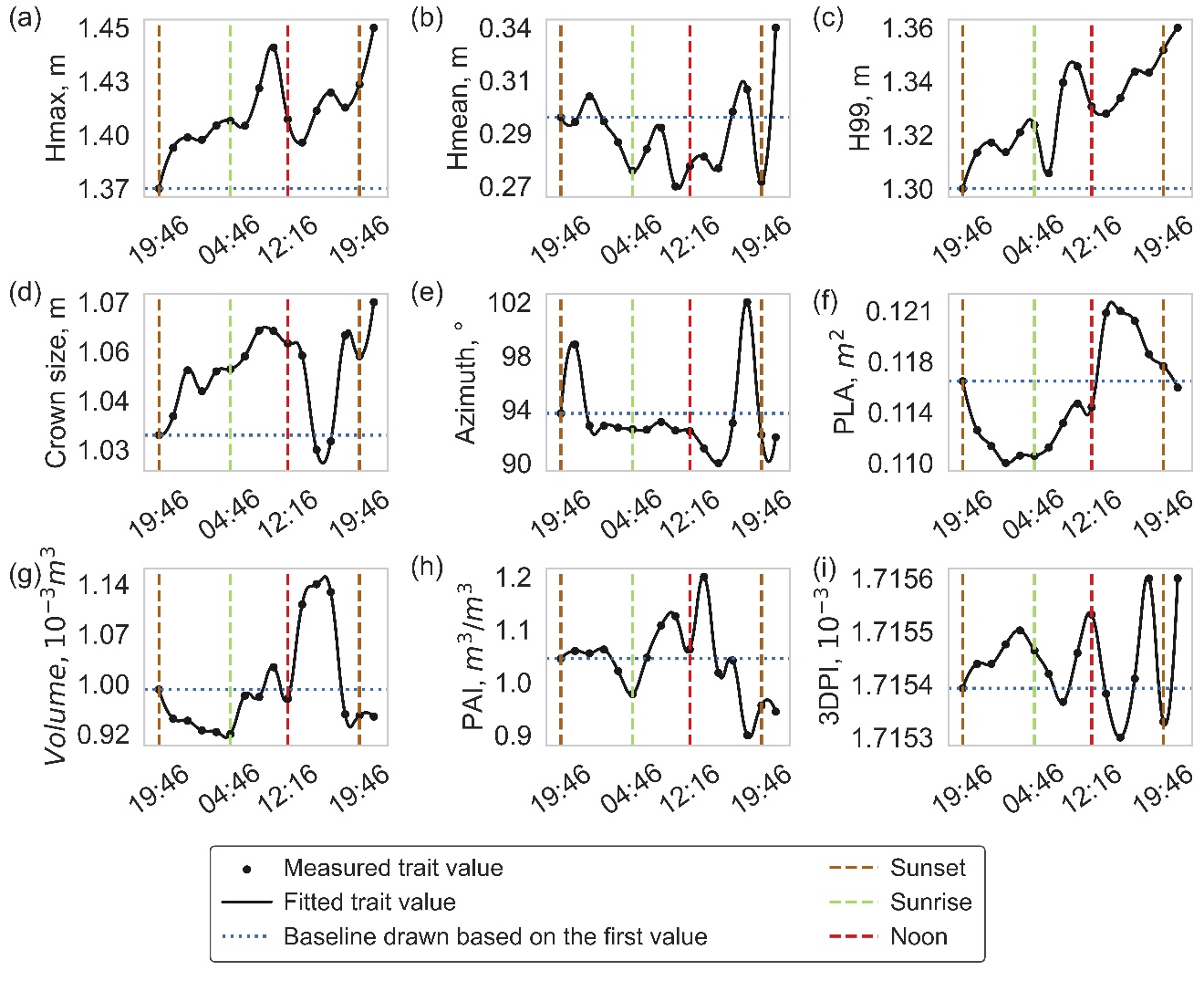


**Figure 2.** Circadian rhythms mixed with self-growing in structural traits of maize at the individual plant level under standard conditions, including (a) Hmax, (b) Hmean, (c) H99, (d) Crown size, (e) Azimuth, (f) PLA, (g) Volume, (h) PAI, and (i) 3DPI. The x-axis is the measured moments from June 22 to June 23, 2019. Each point indicated an averaged trait value of the selected 10 individual plants.


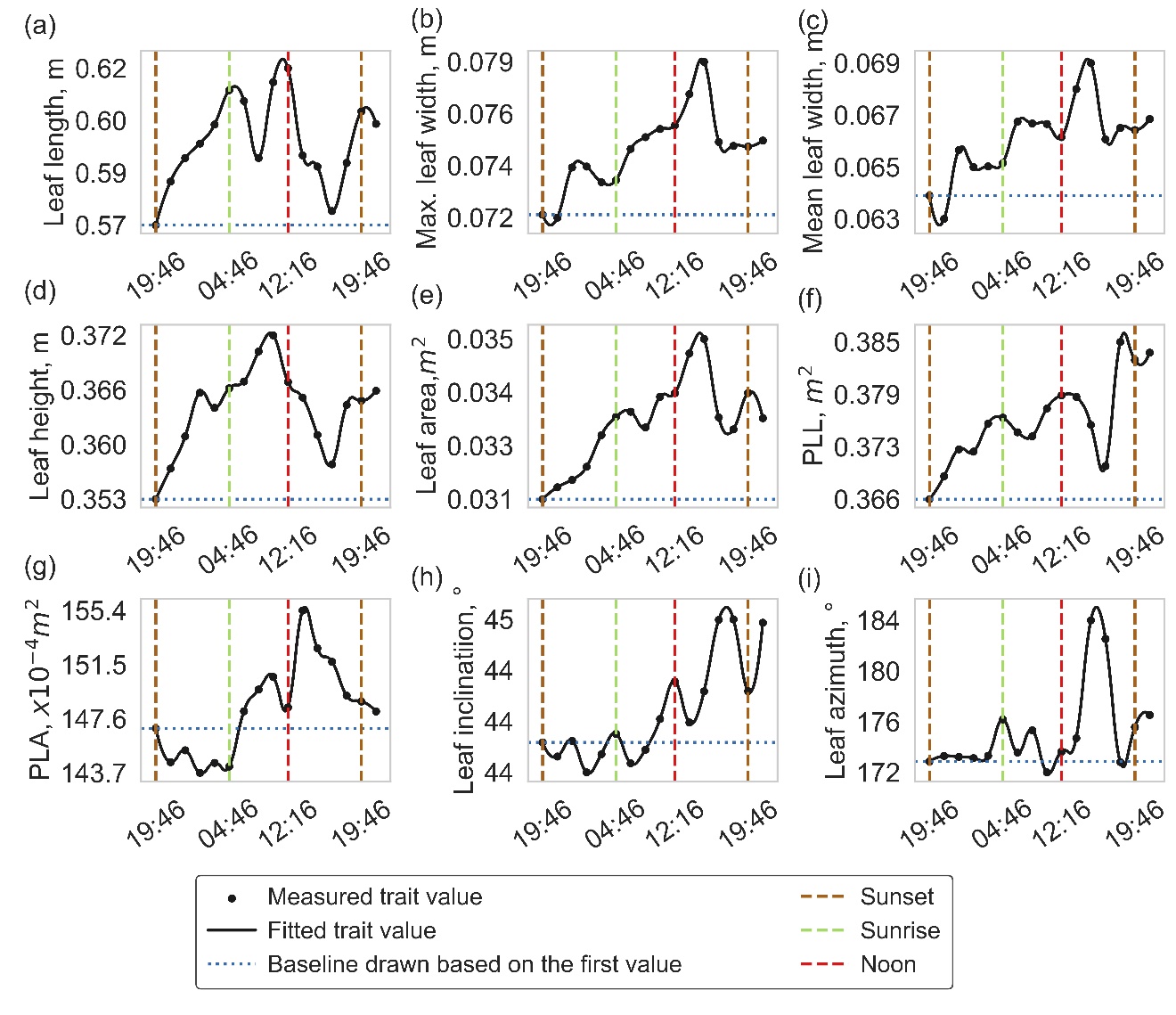


**Figure 3.** Circadian rhythms mixed with self-growing in structural traits of maize at the leaf level under standard conditions, including (a) Leaf length, (b) Max. leaf width, (c) Mean leaf width, (d) Leaf height, (e) Leaf area, (f) PLL, (g) PLA, (h) Leaf inclination, and (i) leaf azimuth. The x-axis is the measured moments from June 22 to June 23, 2019. Each point indicated an averaged trait value of the selected 109 leaves.


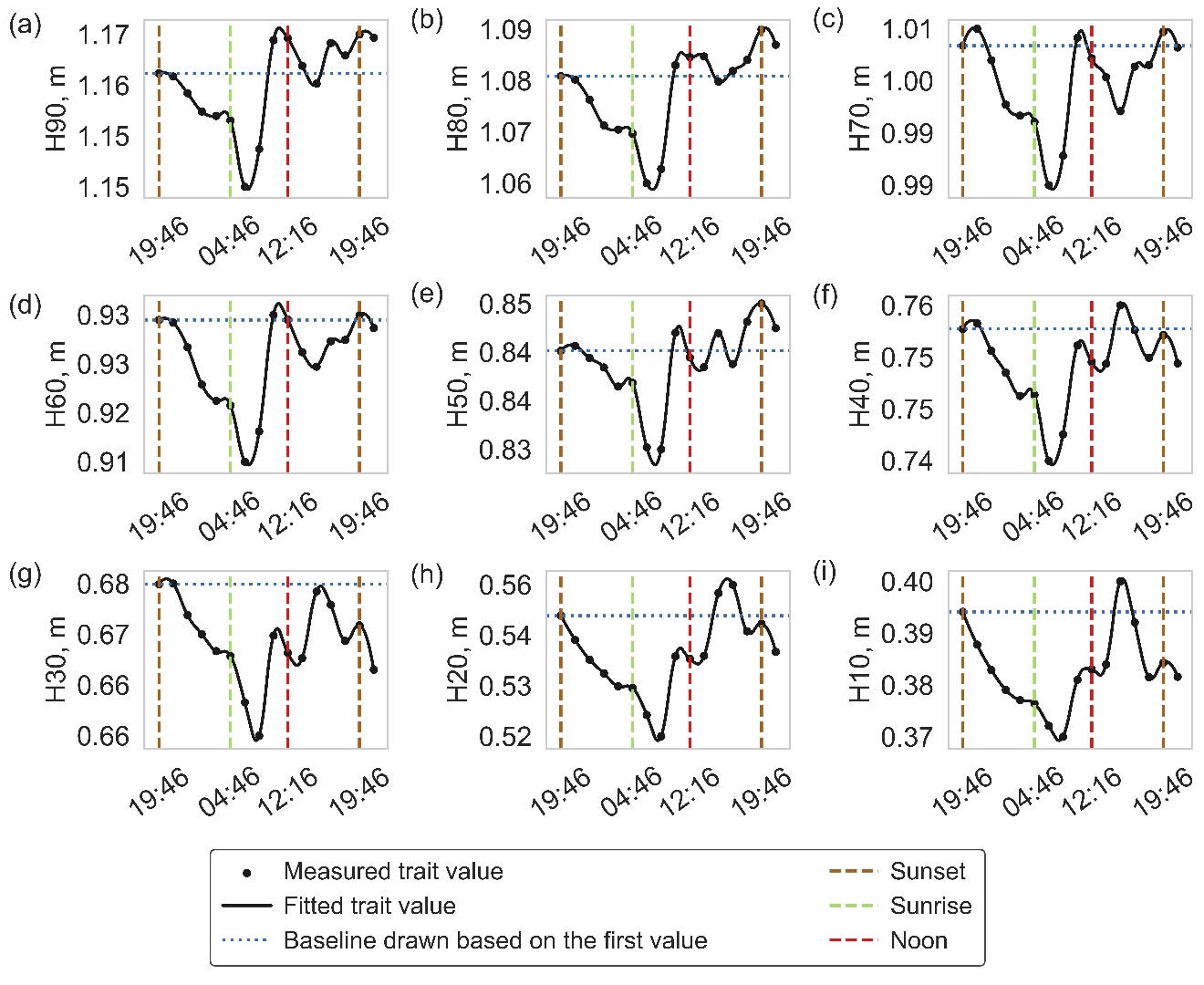


**Fig. 4** Circadian rhythms in structural traits of maize at the individual plant level under standard conditions, including (a) H90, (b) H80, (c) H70, (d) H60, (e) H50, (f) H40, (g) H30, (h) H20, (i) H10. The x-axis is the measured moments from June 22 to June 23, 2019. Each point indicated an averaged trait value of the selected 10 individual plants.


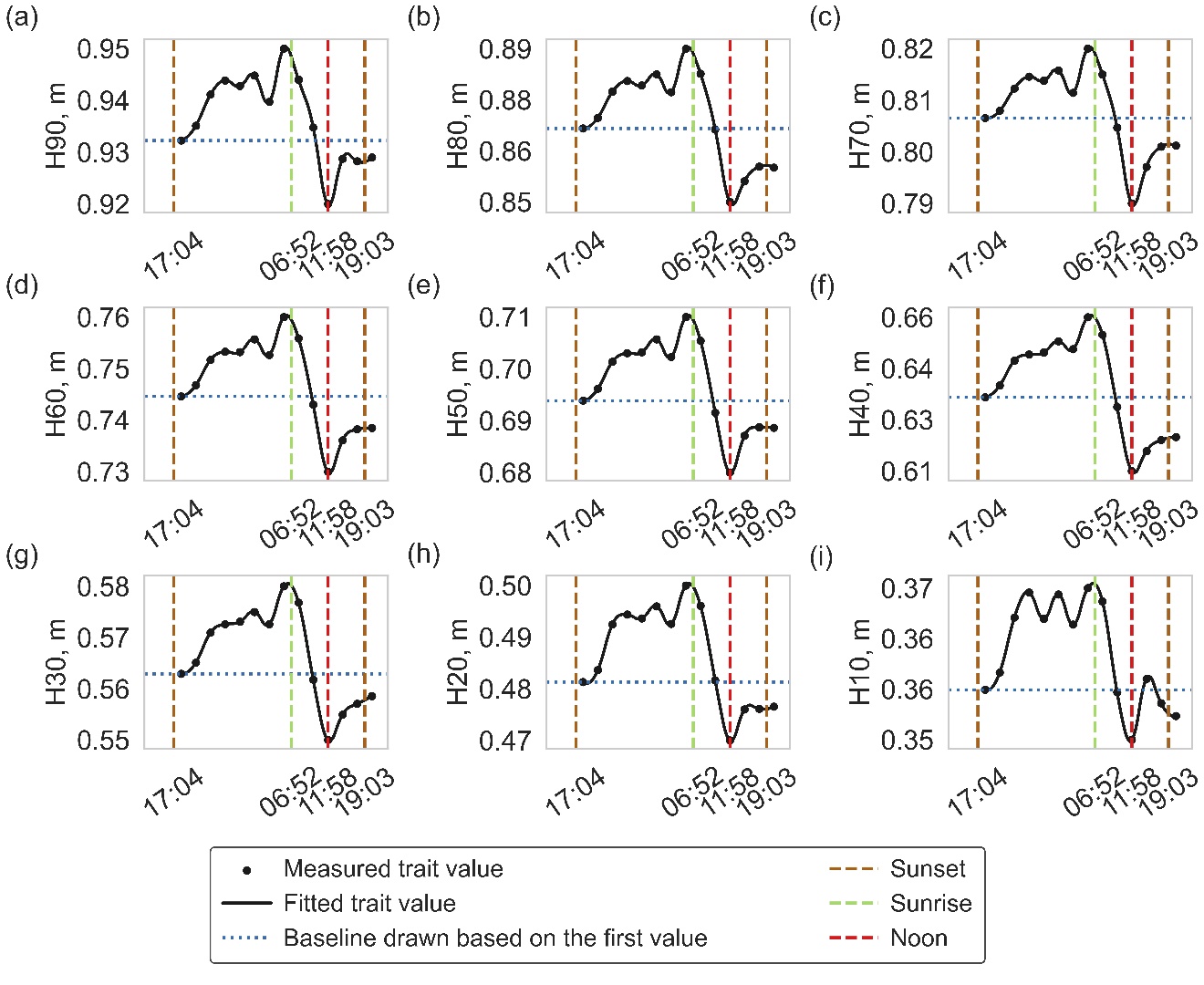


**Fig. 5** Circadian rhythms in structural traits of maize at the individual plant level under cold stress, including (a) H90, (b) H80, (c) H70, (d) H60, (e) H50, (f) H40, (g) H30, (h) H20, (i) H10. The x-axis is the measurement moment from Nov. 9 to Nov. 10, 2019. Each point indicated an averaged trait value of the selected 10 individual samples.
